# Chirality and width effects on elastic and fracture properties of nanoribbons with coronene edges

**DOI:** 10.1038/s41598-026-55137-0

**Published:** 2026-05-26

**Authors:** José M. De Sousa, Marcelo L. Pereira Junior, Douglas S. Galvão, Luiz A. Ribeiro Junior, Alexandre F. Fonseca

**Affiliations:** 1 Instituto Federal de Educação, Ciência e Tecnologia do Piauí (IFPI), Campus São Raimundo Nonato, Primavera, São Raimundo Nonato, Piauí 64770-000 Brazil; 2 College of Technology, Department of Electrical Engineering, University of Brasília (UnB), Brasília, Federal District 70910-900 Brazil; 3 Universidade Estadual de Campinas (UNICAMP), Instituto de Física Gleb Wataghin, Departamento de Física Aplicada, Campinas, São Paulo 13083-859 Brazil; 4 Center for Computing in Engineering and Sciences (CCES), Campinas, São Paulo 13083-859 Brazil; 5 Institute of Physics, University of Brasília (UnB), Brasília, Federal District 70910-900 Brazil; 6 Computational Materials Laboratory (LCCMat), Institute of Physics, Brasília, Federal District 70910-900 Brazil

**Keywords:** Reactive molecular dynamics, Graphene nanoribbon, Coronene nanoribbon, Mechanical properties, Fracture pattern, Chemistry, Materials science, Nanoscience and technology, Physics

## Abstract

Coronenes are a class of polycyclic aromatic hydrocarbons consisting of fused benzene rings arranged in a specific pattern. The structure of coronenes can vary depending on the number of fused benzene rings, with the most common form being hexabenzocoronene, which consists of six benzene rings fused in a circular arrangement. In 2012, coronene-based nanoribbons (CNRs) were successfully synthesized. Although several properties of CNRs have already been studied, a complete characterization of the mechanical properties of CNRs and the effect of the edges are lacking. This study examines how the width and chirality of CNRs affect their mechanical properties under tensile stress. Nanoribbons of rectangular shape and the same width as the CNRs are also studied for the determination of the edge effects on these properties. CNRs of different widths are analyzed, from which the elastic constants are obtained. Results indicate a transition in the mechanical properties from quasi-1D to 2D systems, with the most significant change observed between the narrowest and second-narrowest CNRs considered here. The peak stress is approximately 40 GPa larger for AC-CNRs than for ZZ-CNRs. The response to strain also differs between CNRs with armchair (AC-CNR) and zigzag (ZZ-CNR) edges due to variations in bonding orientations, with AC-CNRs sustaining larger strains than ZZ-CNRs. Fracture patterns are similar for all widths except for the narrowest nanoribbons. Furthermore, the elastic constants of CNRs exhibit an increase with width, approaching the graphene value of 1 TPa, as expected. However, an interesting trend of the Young’s modulus of CNRs with increasing width is observed: the first five AC-CNRs of smallest widths present larger Young’s moduli than those of the first five ZZ-CNRs of smallest widths, and invert for the CNRs with larger values of width. This result is then discussed in terms of the differences in the shape of the edges between AC and ZZ CNRs.

## Introduction

The synthesis of carbon nanotubes^1^ and graphene^2^ has become a milestone in the advancement of nanoscience and nanotechnology^3–8^. Notably, graphene has shown itself to be a remarkable structure^9^. Graphene is a lightweight, one-atom-thick structure whose atoms occupy a densely packed hexagonal network. This gives it unique electronic, mechanical and thermal properties^10^. It exhibits metallic properties and remarkable electronic mobility, with an average value of $2 \times 10^{5}$ cm$^{2}/(\textrm{V}\cdot \textrm{s})$^11,12^. Moreover, graphene displays unparalleled mechanical strength, with a measured value of 55 N/m (130 GPa), and Young’s modulus between 1.0 and 1.1 TPa^13–15^.

Notwithstanding these remarkable properties, the absence of an electronic band gap in pristine graphene imposes constraints on its practical applications in nanoelectronics, including digital transistors^16^. One potential solution to this challenge lies in the exploration of alternative two-dimensional materials with non-zero electronic band gaps that exhibit characteristics analogous to those of graphene^17^. An alternative approach is to modify some of the properties of graphene by means of chemical or physical functionalisation. Graphene oxide^18^, chlorographene, fluorographene, and graphane^19^ are examples of graphene band gap opening by functionalisation. A third approach to achieving a non-zero band gap in graphene involves the cutting and patterning of graphene into nanoribbons with straight^20–22^ or intricate edge structures^23^. This approach can facilitate the design and engineering of nanocircuits^24–26^.

In 2012, Fujihara et al.^27^ reported the successful synthesis of one of these cut and patterned graphene nanoribbons: the so-called coronene-based nanoribbons (CNRs). Its synthesis is achieved through the dimerization of precursors of coronene and dicoronylene ($C_{48}H_{20}$) within carbon nanotubes (CNTs). They showed that, in coronene oligomers, the energies of the excited states decrease with the number of coronene units, and the Raman peak profile in the 1300-1350 cm$^{-1}$ region changes accordingly. Later, in 2016, Barzegar et al.^28^ reported the synthesis of CNRs inside boron nitride nanotubes.

Since then, a few studies about the physical properties of CNRs have been reported in the literature. Verberck, Okazakic, and Tarakina^29^ conducted a study on the packing of coronenes inside CNTs. Monte Carlo simulations were employed to investigate the effect of CNT diameter on the formation of ordered stacks of coronenes or disordered arrangements that enable their polymerisation. Using density-functional tight-binding method, Mandal, S. Sarkar and P. Sarkar^30^ have corroborated the existence of an optimal diameter of the CNT, inside which the encapsulation of the CNR is energetically favourable. Additionally, it was demonstrated that systems formed by CNRs inside CNTs have the potential to be utilised for the storage of hydrogen gas. De Aguiar et al.^31^ employed a combination of spin-polarized tight-binding and density functional theory (DFT) to investigate the electronic and magnetic structures of non-CNT-encapsulated CNRs. They showed that the band gaps of distinct CNRs depend on different magnetic states, thereby rendering them suitable for the development of spintronics. In 2015, Talyzin, Anoshkin, and Nasibulin^32^ empirically demonstrated that elevated temperature (approximately 650 $^{\circ }\hbox {C}$) annealing of CNRs inside CNTs significantly impacts their structural integrity. The electronic structures of CNRs formed by joining coronenes through six-, five- and four-membered rings were investigated by Narita and Okada^33^ using DFT methods. They showed that the configuration involving six-membered rings is the most stable, while the CNR with a five-membered ring exhibited a substantial indirect gap. The enhancement of photoluminescence in CNR-filled nanotubes has been demonstrated by Chernov et al.^34^ Pereira Júnior et al.^35^ have investigated the transport properties of polarons and bipolarons in CNRs. It was shown that zigzag CNRs bipolarons are the only type of CNRs that exhibit mobility. Xie et al.^36^ studied the interfacial excitation energy and charge-carrier transfer CNT-encapsulated CNRs. They showed that there exists an asymmetry in the electron and hole transfer processes between the CNR and the CNT. The electron (hole) transfer from the CNR to the CNT (from the CNT to the CNR) is fast (slow), indicating different concentrations and couplings between acceptor and donor states in the system. Miranda et al.^37^ have shown that the presence of boron and nitrogen heteroatoms in CNRs tunes their electronic properties, inducing a transition from semiconductor to metallic. Furthermore, it was demonstrated that edge substitutions can lead to the emergence of spin-polarized states in CNRs. Although these studies are important for a deeper understanding and applications of CNRs, a significant lacuna remains in the literature on their mechanical properties.

It should be stressed that although CNRs share some topological similarities with graphene nanowiggles^38,39^, they constitute a separate class of nanostructures.

In this work, we examine an important property required for the reliable use of these structures in electronic applications: the elastic properties and fracture patterns of armchair (AC-CNR) and zigzag (ZZ-CNR) coronene-based nanoribbons. A detailed analysis of these features has been conducted, wherein various ribbon lengths and widths were considered. The mechanical properties of straight or rectangular graphene nanoribbons (henceforth referred to as “RNRs”) of similar length and width were also studied in order to provide a comparative study and determine the effects of the edges on the mechanical properties of CNRs. The computational protocol employed here makes use of fully atomistic reactive (ReaxFF) molecular dynamics (MD) simulations. As expected, it was observed that as the system width increases, Young’s modulus approaches the graphene value. Nevertheless, the impact of the edge geometry on the overall mechanical properties of CNRs is only pertinent to CNRs of a limited width.

## Results and discussion

As mentioned above, in order to explore the mechanical properties and fracture patterns of CNRs, classical reactive MD simulations were carried out. The present investigation encompassed CNRs of various widths and chiralities (armchair and zigzag). Regular (rectangular) graphene nanoribbons (RNRs) of equivalent length and width to the CNRs examined in this study were also studied for the purpose of comparison and to determine the effects of the edges on the stress-strain characteristics of CNRs. A total of 25 distinct CNRs (and 24 RNRs) were simulated, with nanoribbon widths ranging up to approximately 100 Å. For comprehensive reference, the dimensions, chirality, and number of atoms per supercell for each of the CNRs (RNRs) under investigation, see Tables 1 and S1 of Supporting Information (SI). These simulations provide valuable insights into the mechanical properties of CNRs and their fracture patterns.

**Table 1:** Information about the simulated CNRs: width (Å) and number of atoms.

System	Width (Å)	Number of atoms
AC-CNR-W$_{1}$	9.28	384
AC-CNR-W$_{2}$	16.75	672
AC-CNR-W$_{3}$	24.04	960
AC-CNR-W$_{4}$	31.47	1248
AC-CNR-W$_{5}$	38.88	1536
AC-CNR-W$_{6}$	46.21	1824
AC-CNR-W$_{7}$	52.64	2112
AC-CNR-W$_{8}$	60.96	2400
AC-CNR-W$_{9}$	67.39	2688
AC-CNR-W$_{10}$	74.77	2976
AC-CNR-W$_{11}$	82.12	3264
AC-CNR-W$_{12}$	89.62	3552
AC-CNR-W$_{13}$	96.89	3840
ZZ-CNR-W$_{1}$	14.42	448
ZZ-CNR-W$_{2}$	22.62	784
ZZ-CNR-W$_{3}$	30.99	1120
ZZ-CNR-W$_{4}$	39.43	1456
ZZ-CNR-W$_{5}$	47.89	1792
ZZ-CNR-W$_{6}$	56.38	2128
ZZ-CNR-W$_{7}$	64.87	2464
ZZ-CNR-W$_{8}$	72.27	2800
ZZ-CNR-W$_{9}$	80.77	3136
ZZ-CNR-W$_{10}$	90.37	3472
ZZ-CNR-W$_{11}$	98.88	3808
ZZ-CNR-W$_{12}$	106.29	4144

**Fig. 1:**
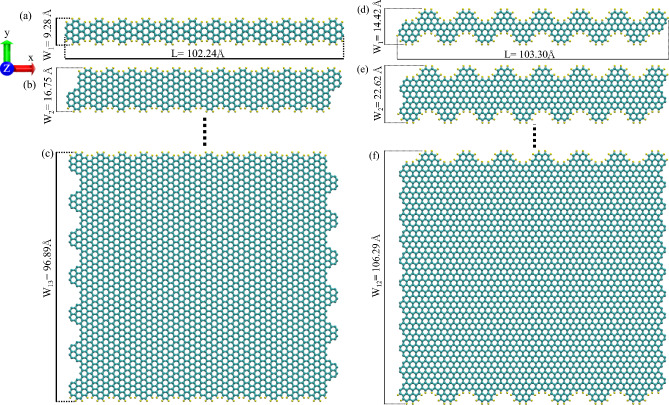
Structure of AC (left panel) and ZZ (right panel) CNRs. The supercells used in the simulations have a length of approximately 102.24 Å (103.30 Å) and consist of 12 (14) coronene-like blocks in AC (ZZ) CNRs. (**a**) The thinnest AC-CNR, denoted as AC-CNR-W$_{1}$. (**b**) The AC-CNR-W$_{2}$ system corresponds to two AC-CNR-W$_{1}$ structures that are fused laterally. (**c**) The AC-CNR-W$_{13}$ system corresponds to 13 AC-CNR-W$_{1}$ structures that are laterally fused. (**d**–**f**) represent the same configurations but for ZZ CNRs. The carbon atoms are represented in cyan color, while the hydrogen atoms are shown in yellow.

See the text for the nomenclature and Fig. 1 for the geometric details of the structures.

Figure 1 displays the armchair (AC) and zigzag (ZZ) structures. Each AC-CNR (ZZ-CNR) is denoted as “AC-CNR-W$_{N}$” (“ZZ-CNR-W$_{N}$”) with “W” representing the width and *N* an index ranging from 1 to 13 (from 1 to 12). The smallest widths of AC- and ZZ-CNRs are shown in Figs. 1a and d, respectively. Wider AC and ZZ nanoribbons of width W$_N$ are created by transversely connecting *N* edge-dehydrogenated units of the AC-CNR-W$_{1}$ and ZZ-CNR-W$_{1}$ structures, respectively. For example, the AC-CNR-W$_{2}$ system consists of two AC-CNR-W$_{1}$ units fused transversely. The choice of *N* chirality is based on the observation that the AC-CNR-W$_{13}$ and ZZ-CNR-W$_{12}$ structures exhibit nearly square shapes. Figures S1 and S2 show some examples of RNRs and details about the structure of CNRs.

**Fig. 2:**
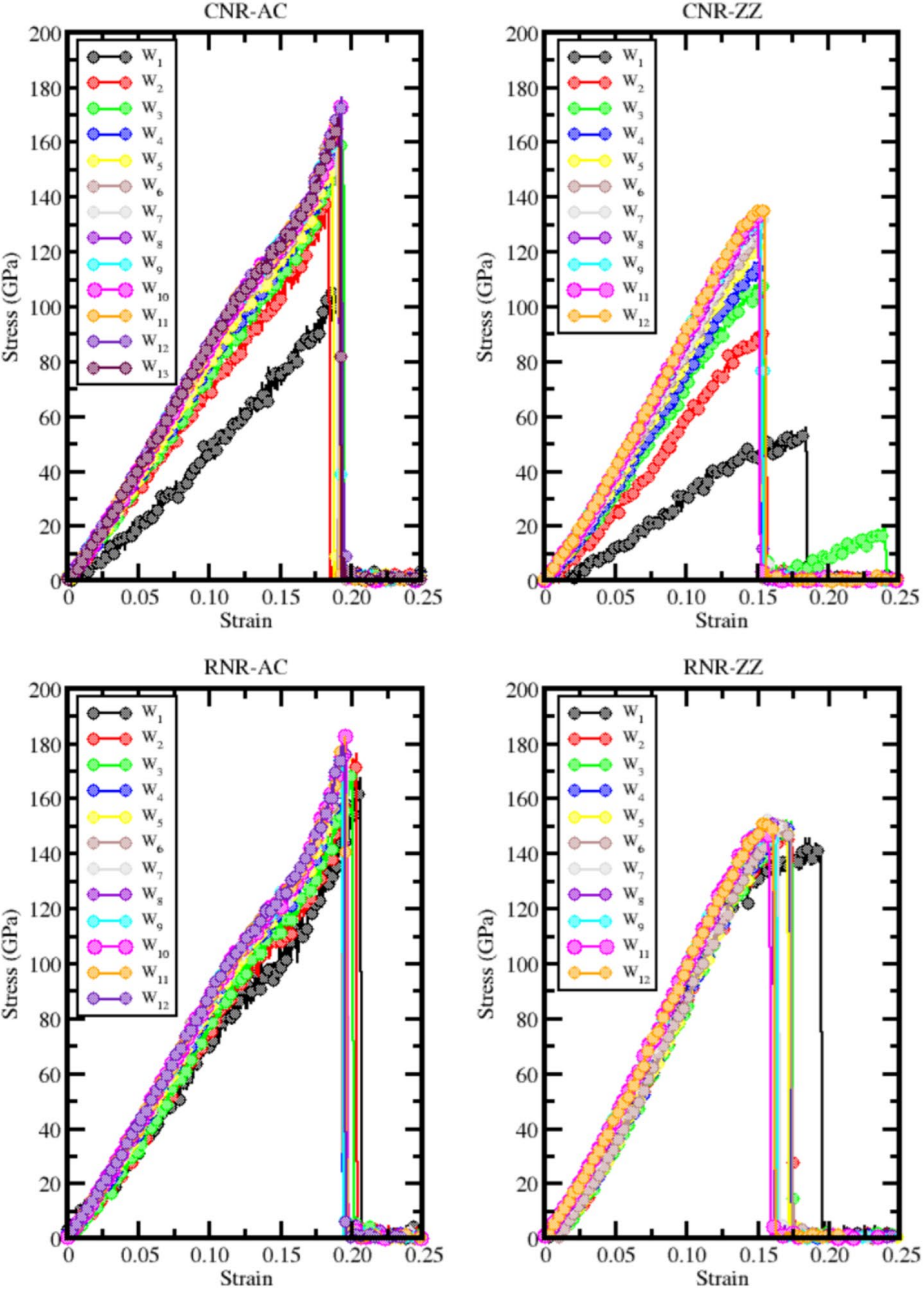
Stress-strain curves. Top: AC-CNRs (ZZ-CNRs) with widths ranging from $W_{1}$ to $W_{13}$ (to $W_{12}$). Bottom: AC-RNRs (ZZ-RNRs) with widths ranging from $W_{1}$ to $W_{12}$ (to $W_{12}$). For further information, refer to Table 1.

Figure 2 displays the stress-strain curves for all AC and ZZ CNRs and RNRs. The curves for each structure type show a convergence trend with increasing width, consistent with the diminishing relevance of edge effects. For RNRs, the convergence is significantly faster, as the various curves for different RNR widths become virtually indistinguishable, resulting in a nearly uniform appearance.

A notable distinction in the stress-strain curves of armchair and zigzag structures is evident in the peak stress (ultimate tensile strength, UTS), value just before the plastic regime of deformations, of AC and ZZ structures. A substantial difference of approximately 40 GPa (30 GPa) is observed between the UTS of AC-CNR-$W_{13}$ and that of ZZ-CNR-W$_{12}$ (between AC-RNR-W$_{10}$ and ZZ-RNR-$W_{12}$). These trends are consistent with the findings of other MD and theoretical studies^40^. With the exception of the ZZ-CNR-W$_{1}$ and ZZ-RNR-W$_1$, the critical strain, $\epsilon _C$, of all ZZ structures is approximately 4 % smaller than that of AC ones (further details below).

With regard to the variations among the same chiral CNRs, it is pertinent to note that the UTS values of the narrowest AC and ZZ CNRs (corresponding to “W$_1$” structures) exhibit a substantial difference of at least *∼* 30 GPa compared to the others.

The UTS of the structures ranging from AC-CNR-W$_2$ to AC-CNR-W$_{13}$ and from ZZ-CNR-W$_3$ to ZZ-CNR-W$_{12}$ exhibit an approximately linear variation. In the specific case of ZZ CNRs, a significant variation in the values of UTS and $\epsilon _C$ is observed between ZZ-CNR-W$_1$ and ZZ-CNR-W$_{2}$ and between ZZ-CNR-W$_2$ and ZZ-CNR-W$_{3}$.

For the ZZ-RNRs, the differences are higher for the critical strain between the ZZ-RNR-W$_1$ and the other structures. These discrepancies suggest a transition from one-dimensional (1D) to two-dimensional (2D) behavior in the structures, which occurs in a manner that differs slightly between armchair and zigzag structures. For AC-CNRs, the transition is quite abrupt, from the AC-CNR-W$_1$ to AC-CNR-W$_{2}$, while for ZZ CNRs, it is less abrupt, from ZZ-CNR-W$_1$ to ZZ-CNR-W$_{3}$.

**Table 2:** Elastic properties of coronene nanoribbons (CNRs) with armchair (AC) and zigzag (ZZ) chirality at room temperature using the reactive classical molecular dynamics method (CMD).

System	$Y_M$ (GPa)	UTS (GPa)	$\epsilon _C$ (%)
AC-CNR-W$_{1}$	486.94 ± 16.19	102.57	19.18
AC-CNR-W$_{2}$	698.44 ± 14.88	140.64	18.52
AC-CNR-W$_{3}$	760.44 ± 13.36	159.85	19.55
AC-CNR-W$_{4}$	799.35 ± 12.69	159.24	19.11
AC-CNR-W$_{5}$	812.31 ± 10.99	153.66	18.77
AC-CNR-W$_{6}$	837.25 ± 2.02	165.34	19.32
AC-CNR-W$_{7}$	849.17 ± 7.42	165.18	19.03
AC-CNR-W$_{8}$	854.05 ± 8.37	164.05	19.05
AC-CNR-W$_{9}$	859.16 ± 14.26	167.49	19.30
AC-CNR-W$_{10}$	863.26 ± 7.10	168.85	19.27
AC-CNR-W$_{11}$	866.19 ± 7.35	172.48	19.45
AC-CNR-W$_{12}$	872.61 ± 5.52	171.18	19.31
AC-CNR-W$_{13}$	872.82 ± 3.02	175.85	19.54
ZZ-CNR-W$_{1}$	318.56 ± 15.57	55.35	18.65
ZZ-CNR-W$_{2}$	604.40 ± 12.12	89.12	15.80
ZZ-CNR-W$_{3}$	719.57 ± 11.46	106.99	15.60
ZZ-CNR-W$_{4}$	767.92 ± 8.69	114.48	15.40
ZZ-CNR-W$_{5}$	805.84 ± 9.57	120.48	15.40
ZZ-CNR-W$_{6}$	846.67 ± 9.10	125.46	15.50
ZZ-CNR-W$_{7}$	862.88 ± 10.24	131.11	15.70
ZZ-CNR-W$_{8}$	880.04 ± 7.74	130.87	15.50
ZZ-CNR-W$_{9}$	893.22 ± 5.50	134.12	15.49
ZZ-CNR-W$_{10}$	900.53 ± 6.18	133.93	15.30
ZZ-CNR-W$_{11}$	909.03 ± 5.59	136.10	15.71
ZZ-CNR-W$_{12}$	918.66 ± 6.91	138.17	15.92

The estimated Young’s Modulus ($Y_{M}$), ultimate tensile strength (UTS), and critical strain ($\epsilon _C$) values for each nanoribbon are presented in the table. The estimated uncertainties in the UTS and $\epsilon _C$ are 5 GPa and 0.05 %, respectively. For an elucidation of the uncertainties, refer to the Methods section.

Table 2 presents the numerical results for the Young’s modulus ($Y_M$), UTS, and $\epsilon _C$ for all AC-CNR and ZZ-CNR systems. The data for RNRs is presented in Table S2 in the SI. In accordance with the preceding discussion on the transition from 1D to 2D, the $Y_M$ values undergo a substantial increase between the W$_1$ to W$_2$ systems, thereby reinforcing the notion of the transition in this particular elastic property. As systems become larger, the rate of increase in $Y_M$ slows, and this trend is consistent across both chiralities, with $Y_M$ of AC-CNR structures converging more quickly with width than that of ZZ-CNR ones. This difference in the convergence of the elastic properties between AC and ZZ CNRs may be attributable to the configuration of the edges of both chiral structures. As shown in Fig. 1, the lateral edges of AC-CNRs exhibit a lesser degree of undulation in comparison to those of ZZ-CNRs. Another observation is that the Young’s moduli of the first five smallest width AC-CNRs are correspondingly larger than those of ZZ-CNRs. This finding indicates a precise inverse relationship between the Young’s moduli of AC and ZZ RNRs. As indicated in Table S2 of the SI, the Young’s moduli of all ZZ-RNRs are higher than those of AC-RNRs. Consequently, the inverted trend that was observed in the five smallest width CNRs can be attributed to the presence of edge undulations. Notably, the Young’s modulus values of the AC-CNR-W$_{13}$ and ZZ-CNR-W$_{12}$ cases are approximately 872 GPa and 919 GPa, respectively, which are comparable to those of graphene (between 1.0 and 1.1 TPa^13–15^). Similar results were obtained for the RNRs (see Table **S2** of SI). The critical strain, $\epsilon _C$, exhibits a relatively consistent pattern across all armchair systems, with an average value of approximately 19%, while in zigzag systems, it is about 4% lower, averaging around 15% (with the exception of the ZZ-CNR-W$_1$ system).

**Fig. 3:**
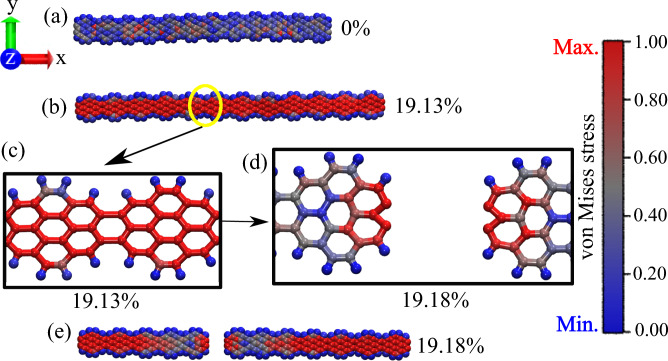
Representative MD snapshots illustrating the tensile stretching process of an AC-CNR with a width of $W_{1} = 9.28$ Å. Please refer to Table 1 for additional information. (**a**) Initial structure of the CNR at 0% strain. (**b**) Structure of the CNR at 19.13% strain. (**c**) A zoomed-in view of the yellow circled region of the structure shown in (**b**), showing some stretched C–C bonds. (**d**) A zoomed-in view of the broken region of the CNR. (**e**) Fractured CNR at *19.18%* strain. The sidebar on the right defines the color coding: red indicates high-stress accumulation, and blue indicates low-stress accumulation.

We have examined the fracture patterns in two extreme scenarios, considering the narrowest and widest structures from armchair and zigzag chiralities. Specifically, Fig. 3 illustrates the fracture pattern of the AC-CNR-W$_1$ system. Panel (a) presents the system under no uniaxial tension. Here, blue and red represent minimum and maximum stress levels, respectively, at each atom, according to the von Mises stress criterion (see methodology section). The greyish pattern of the atoms’ colours of the structure depicted in Fig. 3a indicates the presence of local thermal stresses due to thermal fluctuations, and does not imply that the resultant stress is nonzero. The presence of two atomic types (C and H) in the system is of little consequence, given that hydrogen atoms are confined to the system’s edges and therefore do not significantly contribute to stress. Panel (b) of Fig. 3 depicts the precise “moment” immediately preceding the fracture, at 19.13% strain, where bonds (with the exception of hydrogen atom edges) are highlighted in red, denoting uniform stress throughout the entire chain. In panel (c), the bonds connecting adjacent coronene-like units are emphasised. Subsequently, the system undergoes an abrupt fracture, specifically breaking the bonds between the laterally connected coronene blocks (panel (d)). Finally, Fig. 3e shows the system immediately after fracture, at 19.18% strain.

**Fig. 4:**
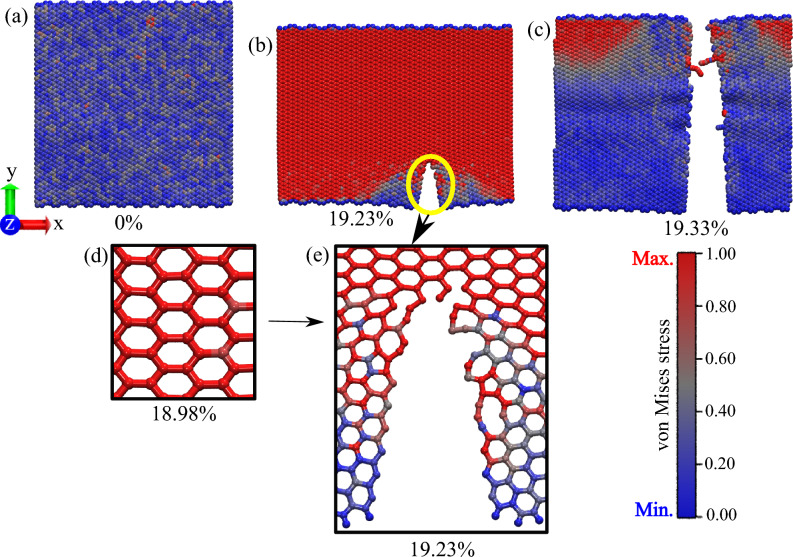
Representative MD snapshots illustrating the tensile stretching process of an AC-CNR with a width of $W_{13} = 96.89$ Å. Please refer to Table 1 for additional information. (**a**) Initial structure of the CNR at 0% strain. (**b**) Structure of the CNR after the crack initiation at 19.23% strain. (**c**) Structure of the CNR after the complete fracture at 19.33% strain. (**d**) and (**e**) Zoomed views of the structure before and during the rupture, highlighting the stretch and break of C–C bonds at 18.98% and 19.23% strains, respectively. The sidebar on the right defines the color coding: red indicates high-stress accumulation, and blue indicates low-stress accumulation.

Figure 4 illustrates the fracture pattern of the widest AC-CNR studied, the AC-CNR-W$_{13}$ (approximately 96.89 Å wide). Although the rupture starts at one of the edges, the comparison of $Y_M$, UTS and $\epsilon _C$ between the AC-CNR-W$_{13}$ (Table 2) and AC-RNR-W$_{12}$ (Table **S2** of SI) structures reveals no significant differences, indicating the convergence of the mechanical properties of the CNRs with those of graphene as the width increases. Furthermore, the rupture of the widest rectangular nanoribbon also initiated at the edges (see Fig. S5 of SI). Panel (d) of Fig. 4 shows the system at 18.98% strain, exhibiting a significant resemblance to graphene. In reality, a minor edge effect is observed on the fracture pattern of the CNRs. In all AC (and also in ZZ as shown below) CNR systems, the integrity of the coronene-like parts of the edges is observed. In other words, the rupture invariably originates from the region between these coronene parts. Another observation is the formation of linear atomic chains (LACs) of carbon atoms that occur after the abrupt drop in stress during fracture.

**Fig. 5:**
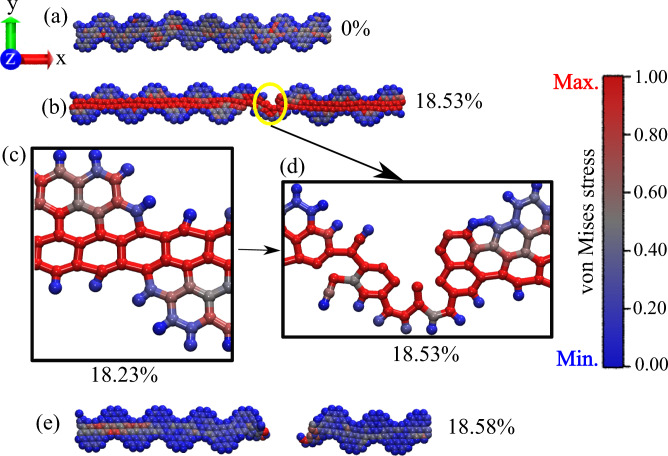
Representative MD snapshots illustrating the tensile stretching process of a ZZ-CNR with a width of $W_{1} = 14.42$ Å. Please refer to Table 1 for additional information. (**a**) Initial structure of the CNR at 0% strain. (**b**) Structure of the CNR after the crack initiation at 18.53% strain. (**c**) A zoomed view of the C–C chemical bonds at 18.23% strain, just before the first bond breaking. (**d**) A zoomed view of the yellow circled region of the structure shown in panel (**b**) showing the first broken C–C bonds. (**e**) Fractured CNR at 18.58% strain. The sidebar on the right defines the color coding: red indicates high-stress accumulation, and blue indicates low-stress accumulation.

Figure 5 illustrates the fracture pattern of the narrowest width system in zigzag chirality. Panel (a) displays the initial state of the balanced system without any strain. Panel (b) shows the onset of system fracture, occurring at 18.53% strain. Panel (c) illustrates the system prior to fracture, revealing strained bonds along the region parallel to the tensile forces. Panels (b) and (c) illustrate the portions of the system that experience lower tension. The central line of hexagons, aligned with the tensile strain direction, constitutes the primary stressed region of this CNR. Figure 5d is a magnification of the rupture region shown in panel (b). Due to different edge topologies between the AC-CNR-W$_1$ and ZZ-CNR-W$_1$ systems, in contrast to the armchair counterpart, once the first C–C bond breaks at one edge, the remaining structure resembles a bent system that first stretches and then fully breaks under continued application of tensile strain. That is similar to what is seen in the fracture patterns of graphene nanowiggles^39^. This elucidates the reason for the particular advantage of the ZZ-CNR-W$_1$ structure in withstanding a higher relative amount of critical strain than the others (18.65 % versus *∼*15 % on average for all other ZZ-CNRs). As previously discussed, the degree of undulation exhibited by the ZZ-CNR-W$_1$ structure was found to enable the bending behavior during the crack propagation. For all other AC and ZZ-CNRs, the edges exerted negligible influence on the fracture patterns, and the observed difference in the average critical strain, $\epsilon _C$, can be attributed to the known propensity for fracture along the zigzag direction in graphene^41,42^. Finally, Fig. 5e portrays an instant after fracturing at 18.58% strain, indicating the same abrupt fracture behavior as was observed in AC structures. When considering wider widths, the differences between zigzag and armchair chirality systems are not significant.

**Fig. 6:**
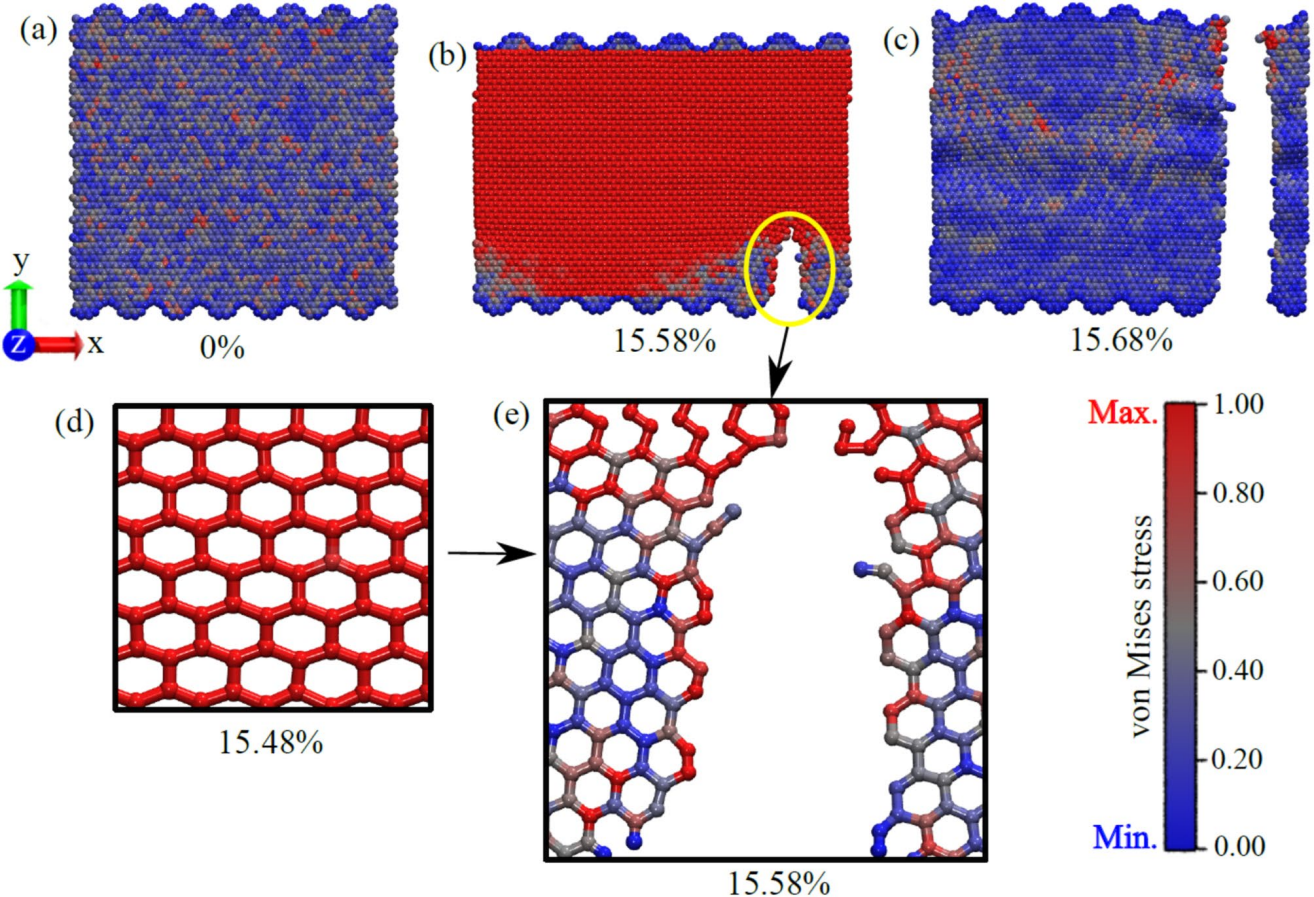
Representative MD snapshots illustrating the tensile stretching process of a ZZ-CNR with a width of $W_{12} = 106.29$ Å. Please refer to Table 1 for additional information. (**a**) Initial structure of the CNR at 0% strain. (**b**) Structure of the CNR after the crack initiation at 15.58% strain. (**c**) Structure of the CNR after the complete fracture at 15.68% strain. (**d**) and (**e**) Zoomed views of the structure before and during the rupture, highlighting the stretch and break of C–C bonds at 15.48% and 15.58% strains. The right sidebar uses color to indicate stress accumulation: red indicates high-stress areas, and blue indicates low-stress regions.

Figure 6 displays significant snapshots of the ZZ-CNR-W$_{12}$ structure during the tensile strain process. In panel (a), the system is observed in its pristine state, devoid of any applied strain. Panel (d) provides a closer view of a region away from the edges, highlighting its resemblance to graphene. Panels (b) and (e) depict the system at the onset of the fracture process, displaying similarities to the same process happening to the armchair large-width systems. The fracture initiation and completion strains are determined to be 15.58% and 15.68%, respectively, thereby indicating the brittleness of the fracture.

An interesting additional observation, based on Tables 2 and **S2**, is that the differences in UTS values between large-width AC-RNRs and AC-CNRs are less than half those between ZZ-RNRs and ZZ-CNRs, indicating a possible residual effect of edge effects on this property.

**Fig. 7:**
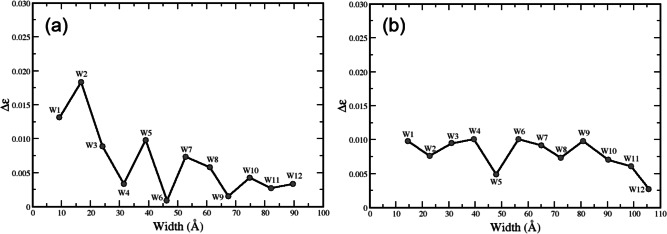
Graphical representations of critical strain differences between CNRs and RNRs of similar width as functions of the original width. Panels (**a**) and (**b**) show results for AC and ZZ structures, respectively.

Finally, we analysed the differences in the stress-strain curves between CNRs and RNRs of the same chirality and similar width. The stress-strain curves of CNRs and RNRs of the same or similar width are plotted together in Figs. S7 and S8 for AC and ZZ structures, respectively. These figures provide an overview of the differences in the mechanical behaviors between CNRs and RNRs, for each width. The sequence of panels in these figures delineates the manner in which the stress-strain differences between CNRs and RNRs are contingent on their width values. They demonstrated that, as the width increases, the stress-strain curves tend to become indistinguishable from one another. Furthermore, the peak stress (UTS) and the critical strain values ($\epsilon _C$) tend to become equivalent as the width increases. However, a divergence in the convergence trends between AC and ZZ structures has been observed. It is evident from Figs. S7 and S8 that, for the values of $\epsilon _C$, the ZZ structures exhibit heightened sensitivity to the coronene-like edges in comparison to the AC ones. The differences in $\epsilon _C$, $\Delta \epsilon _C$, between CNRs and RNRs of similar width values were quantified, and the graphics depicted in Fig. 7 were plotted. Panels (a) and (b) show $\Delta \epsilon _C$ as a function of the nominal width for AC and ZZ structures, respectively. It shows that for AC structures, convergence to the similarity between CNRs and RNRs is faster than for ZZ structures.

## Methods

The simulations performed in this study used the interatomic reactive force field ReaxFF^43^, which integrates quantum-chemical force fields with empirical binding-energy terms. The numerical integration of Newton’s equations was performed within the LAMMPS (Large-scale Atomic/Molecular Massively Parallel Simulator) code^44^. The set of parameters employed in the simulations was developed by Mueller, van Duin and Goddard III^45^.

ReaxFF derives its parameters from experimental data and/or first-principles calculations, thus rendering it a versatile tool with which to investigate diverse nanostructures. This force field enables the simulation of the dynamic formation and dissociation of chemical bonds, accurately reflecting the evolving connectivity of the nanostructures as a function of the atomic positions. ReaxFF has been successfully employed to study the mechanical and thermal properties of several one-dimensional^46–51^ and two-dimensional (2D)^52–62^ nanomaterials. Furthermore, the convergence of the results for the Young’s moduli of our nanoribbons with increasing width to that of graphene (between 1.0 and 1.1 TPa^13–15^) indicates that the choice of potential and force field is validated.

All reactive classical molecular dynamics simulations are initiated with energy minimization of all structures prior to the application of tensile strain. The minimization algorithm employed in all computations is Style *cg*, which is the “Polak-Ribière” version of the conjugate gradient (CG) algorithm. The convergence criteria that were adopted in the energy minimization processes were as follows: the energy must be less than $1 \times 10^{-4}$ (unitless) and the forces must be less than $1 \times 10^{-6}$ Kcal.mol$^{-1}$Å$^{-1~}$^63–65^. All structures are subjected to periodic boundary conditions along the direction of strain.

All structures are thermalized at 300 K and kept at a null pressure for 100 ps using a Nosé-Hoover pressure barostat^66^ to achieve a balanced nanostructure without any residual stresses. Subsequently, the nanostructures undergo thermalization for an additional 100 ps in the canonical ensemble NVT (constant number of particles, volume, and temperature). During the stretching process of the CNRs, the canonical ensemble NVT is maintained at a temperature of 300 K. The thermal equilibration is controlled by the Nosé-Hoover thermostat^67^. A time step of 0.05 fs was employed to ensure accurate numerical integration of the temporal equations of motion, while maintaining a constant engineering strain rate of $\vartheta = 10^{-6}$ fs$^{-1}$.

This value of strain rate has been successfully used by some of us^47,48^ and others^49,68^ in stress-strain studies of carbon structures. In particular, Jensen, Wise, and Odegard^68^ have demonstrated that the outcomes of stress-strain simulations in carbon nanostructures are not sensitive to strain rate, provided it is less than 2.2 ns$^{-1}$. The length, *L*, of the structures will undergo a simple change described by $L(t) = L_0(1 + \vartheta t)$, where $L_0$ represents the initial length of the structure.

The stress tensor of the *i*-th atom is estimated by LAMMPS through the following formula:

1 $$\begin{aligned} \sigma _{ab}=mv_av_b+\frac{1}{2}\sum ^{N_p}_j\left( r_{1a}F_{1b}+r_{2a}F_{2b}\right) \, , \end{aligned}$$

where, *a* and *b* represent any of the *x*, *y* or *z* coordinates, $v_a$ is the *a*-component of the velocity of the *i*-th atom, and the second term represents the virial contribution to the stress on the *i*-th atom due to its interaction with $N_p$ neighbours. Here, $\textbf{r}_j$ (*j=1,2*) are the position vectors of each pair of interacting atoms and $\textbf{F}_j$ is the resultant force on atom *j*.

It is possible to obtain a comprehensive understanding of the stress distribution and fracture behavior during the stretching process of the structures by using the von Mises stress or von Mises criterion ($\sigma _\text {VM}$)^69^. This criterion considers the combined influence of normal and shear stresses exerted on the nanostructure. Mathematically, this can be expressed as follows:

2 $$\begin{aligned} \sigma _\text {VM} = \sqrt{ \frac{(\sigma _{xx}-\sigma _{yy})^2 + (\sigma _{yy}-\sigma _{zz})^2 + (\sigma _{zz}-\sigma _{xx})^2 + 6(\sigma _{xy}^2 + \sigma _{yz}^2 + \sigma _{zx}^2)}{2} }, \end{aligned}$$

where $\sigma _{ab}$, is given by Eq. (1).

To estimate Young’s modulus values, $Y_M$, the linear region of the stress-strain curves up to 1% is used. It is defined as the ratio of the normal stress ($\sigma _{xx}$) to the strain along the *x* direction ($\varepsilon _{x}$). The standard deviation in Young’s modulus is obtained from the fitting of the stress-strain values at this region. The strain is calculated as the relative change in length $(L - L_0)/L_0$, where *L* represents the current length and $L_0$ denotes the initial length of the structures along the periodic axis. The largest uncertainty in the UTS values can be estimated by taking the difference between adjacent points in Fig. 2, close to the maximum value of stress before fracture. The uncertainty in the critical strain can be estimated by the difference in the frames collected to generate the trajectory of the simulations. Each frame was exported every 500 fs of simulation, corresponding to an uncertainty in the critical strain of 0.05 %, at a constant strain rate of $\vartheta = 10^{-6}$ fs$^{-1}$.

The evaluation of von Mises stress and Young’s modulus is a valuable approach to gaining insights into the stress distribution and mechanical properties of CNRs during stretching.

For the purpose of visualisation and analysis of the molecular configurations and stretching dynamics of the structures investigated in this study, we have used the Virtual Molecular Dynamics Visualization (VMD) software^70^. VMD provides a comprehensive suite of tools for the visualisation and comprehension of nanostructures, thereby facilitating a thorough examination of their properties and behaviors. The snapshots visualised with VMD allow for the observation of specific points in time, changes in the structures across different strain values, atomistic configurations along the stretching directions, and bond breakage and formation during plastic deformation. These observations are essential for understanding the origins of structural mechanical failure.

## Conclusions

In summary, this study provides a comprehensive analysis of the mechanical properties of coronene-based carbon nanoribbons (CNRs) under uniaxial tension, focusing on the influence of width and chirality. Furthermore, the mechanical properties of rectangular nanoribbons were generated and studied for the purpose of comparison and to identify the limits to which edge effects become non-significant. These findings enhance our understanding of the mechanical behavior of CNRs and their potential applications in nanoscale devices.

The width and chirality of the CNRs were shown to be crucial factors in their mechanical response. The values of Young’s modulus, ultimate tensile stress (UTS), and critical strains were extracted from MD simulations of tensile strain at a constant strain rate. Despite the observed tendency of the mechanical properties of CNRs to those of graphene with increasing width, a significant influence of the edges, from a mechanical point of view, on the mechanical properties of, at least, small-width zigzag CNRs is evident. The differences between CNRs and RNRs are further evidenced by pronounced changes in stress-strain curves and elastic constants, particularly for the narrowest systems. A comparison of the mechanical properties of CNRs and RNRs as a function of their width reveals two interesting differences. The elastic moduli of the first five smallest-width AC-CNRs are observed to exceed those of the first five smallest-width ZZ-CNRs. For the RNRs, the elastic moduli of AC configurations are always smaller than those of ZZ configurations, a finding that is further supported by the results of a previous study^71^. The second interesting difference is in the relation between the UTS values of the smallest-width CNRs and RNRs. The UTS of the ZZ-CNR-W$_1$ (ZZ-RNR-W$_1$) is smaller (larger) than the UTS of the AC-CNR-W$_1$ (AC-RNR-W$_1$). These results are directly a consequence of the edge topology of CNRs. Understanding the fracture behavior and the influence of the quasi-1D structure on the mechanical properties of CNRs is essential for tailoring their mechanical properties to specific applications.

In circumstances where a device requires the use of small-width nanoribbons of higher elastic modulus, UTS and critical strain, AC-CNRs are the optimal candidates (the five smallest-width AC-CNRs present larger Young’s moduli than the five smallest-width ZZ-CNRs). In cases where the electronic features of ZZ-CNRs are considered more significant than those of AC-CNRs, and the structure or device is to be subjected to substantial tensile strain, the present study indicates that the ZZ-CNR-W$_1$ can withstand as much critical strain as the AC-CNRs. The UTS values of small-width AC-CNRs rapidly approach 160 GPa from the “W$_3$” onward, while those of ZZ-CNRs reach *∼*130 GPa from the “W$_6$” onward. This finding suggests potential applications of the CNRs as stress sensors within the range of 50 to 175 GPa. Therefore, the variations observed in the fracture patterns of the narrowest nanoribbons underscore the necessity for careful consideration of the width-dependent mechanical behavior and fracture characteristics when designing nanoribbon-based devices.

## Supplementary Information

Supplementary Information.

## Data Availability

The datasets utilized and/or examined throughout the present study are accessible upon reasonable request from the corresponding author.
