# Supplementary material for: Chirality and width effects on elastic and fracture properties of nanoribbons with coronene edges
Source: Sci Rep. 2026 May 26;16:24087. doi: 10.1038/s41598-026-55137-0 (PMC13439204; doi:10.1038/s41598-026-55137-0)
Supplement: Supplementary file 1 [file 41598_2026_55137_MOESM1_ESM.pdf]

Supplementary Information for
Chirality and width effects on elastic and fracture properties of nanoribbons with coronene edges

José M. De Sousa,[1] Marcelo L. Pereira Junior,[2] Douglas S. Galvão,[3,4] Luiz A. Ribeiro Junior,[5,6] and Alexandre F. Fonseca[3,*]

[1] *Instituto Federal de Educação, Ciência e Tecnologia do Piauí – IFPI, São Raimundo Nonato, Primavera, 64770-000, São Raimundo Nonato, Piauí, Brazil.*

[2] *University of Brasília, College of Technology, Department of Electrical Engineering, Brasília, Federal District, Brazil.*

[3] *Universidade Estadual de Campinas, Instituto de Física Gleb Wataghin, Departamento de Física Aplicada, 13083-859, Campinas, SP, Brazil.*

[4] *Center for Computing in Engineering and Sciences, State University of Campinas – UNICAMP, Campinas 13083-859, SP, Brazil.*

[5] *Institute of Physics, University of Brasília (UnB), Brasília, Federal District 70910-900, Brazil.*

[6] *Computational Materials Laboratory (LCCMat), Institute of Physics, University of Brasília (UnB), Brasília, Federal District 70910-900, Brazil.*

SUPPORTING INFORMATION

The present document contains supplementary results that were not included in the main manuscript. Snapshots of the structures of rectangular graphene nanoribbons (RNRs) are presented, as well as stress-strain curves of both the RNRs and coronene-based nanoribbons (CNRs) with armchair (AC) and zigzag (ZZ) edges.

Figures S1 and S2 show fully atomic models of examples of armchair and ZZ-RNRs and ZZ-CNRs, respectively. Figs. S3 and S4 show representative MD snapshots of the tensile stretching process of AC and ZZ RNRs, respectively. Tables S1 and S2 presents the information about the geometric features and the mechanical properties of the RNRs simulated in this work.

* Corresponding author: afonseca@ifi.unicamp.br

I. Graphene nanoribbon - RNRs and CNRs

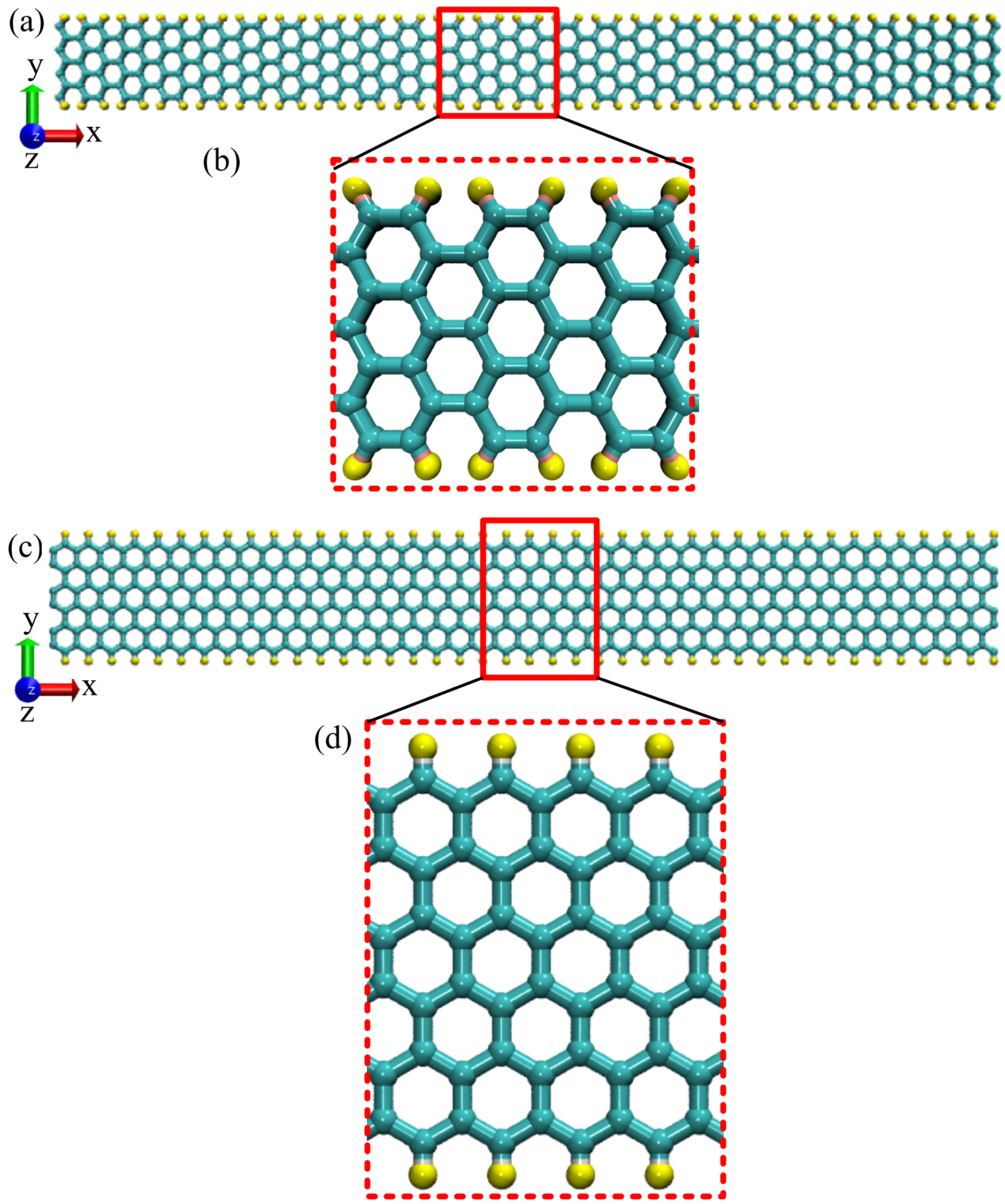

FIG. S1. **Representative atomic models of RNRs with armchair (AC) and zigzag (ZZ) chiralities.** (a) Frontal view in the van der Waals and dynamics bonds representation of an AC-RNR. In (b), a zoomed (red rectangle) region of the RNR shown in panel (a) showing the carbon atoms (cyan color) and hydrogen atoms (yellow color). (c) Frontal view in the van der Waals and dynamics bonds representation of a ZZ-RNR. In (d), a zoomed (red rectangle) region of the RNR shown in panel (c), showing the carbon atoms (cyan color) and hydrogen atoms (yellow color).

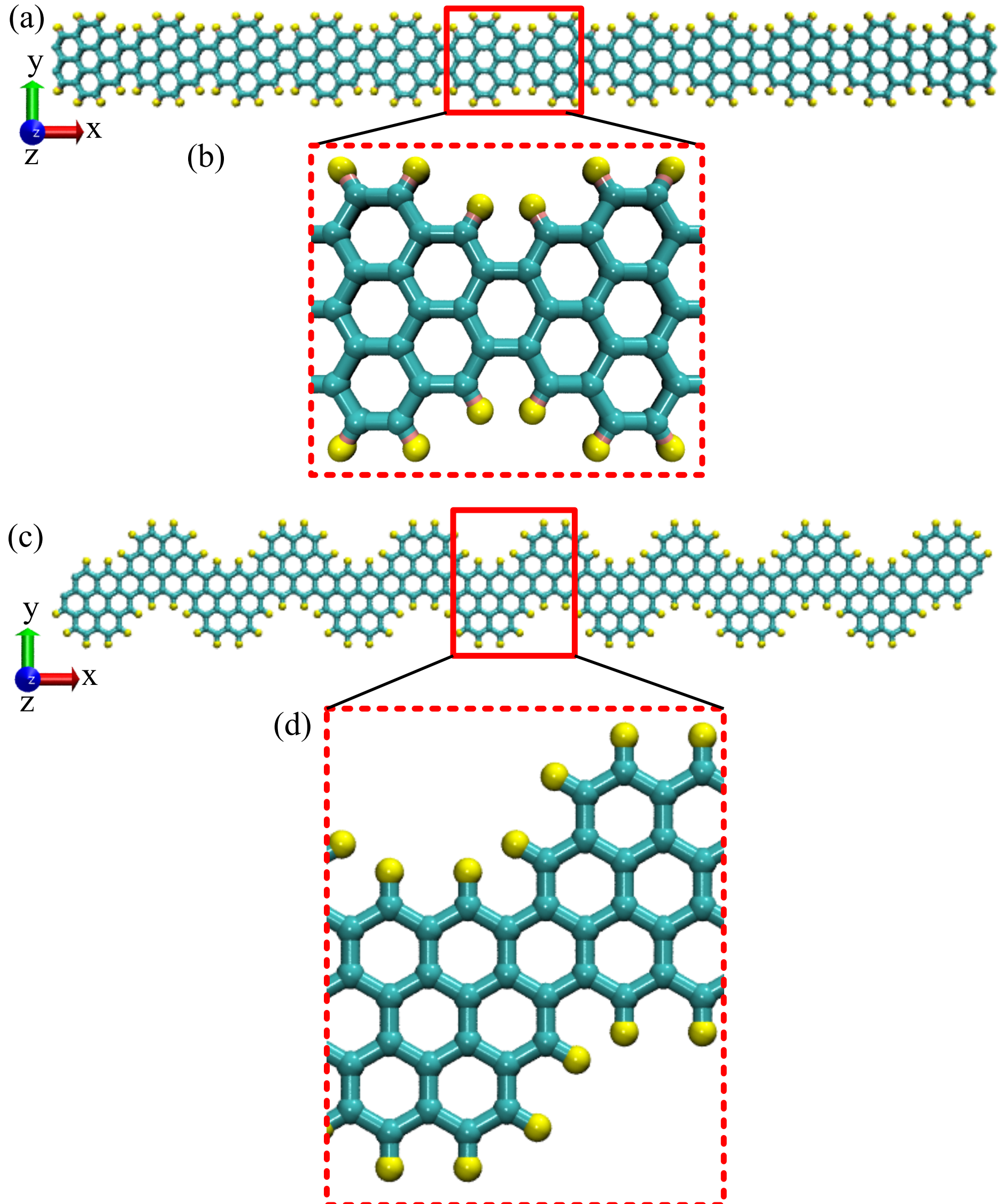

FIG. S2. **Representative atomic models CNRs with armchair (AC) and zigzag (ZZ) chiralities.** (a) Frontal view in the van der Waals and dynamics bonds representation of an AC-CNR. In (b), a zoomed (red rectangle) of a region of the CNR shown in panel (a), showing the carbon atoms (cyan color) and hydrogen atoms (yellow color). (c) Frontal view in the van der Waals and dynamics bonds representation of a ZZ-CNR. In (d), a zoomed (red rectangle) of a region of the CNR shown in panel (c), showing the carbon atoms (cyan color) and hydrogen atoms (yellow color).

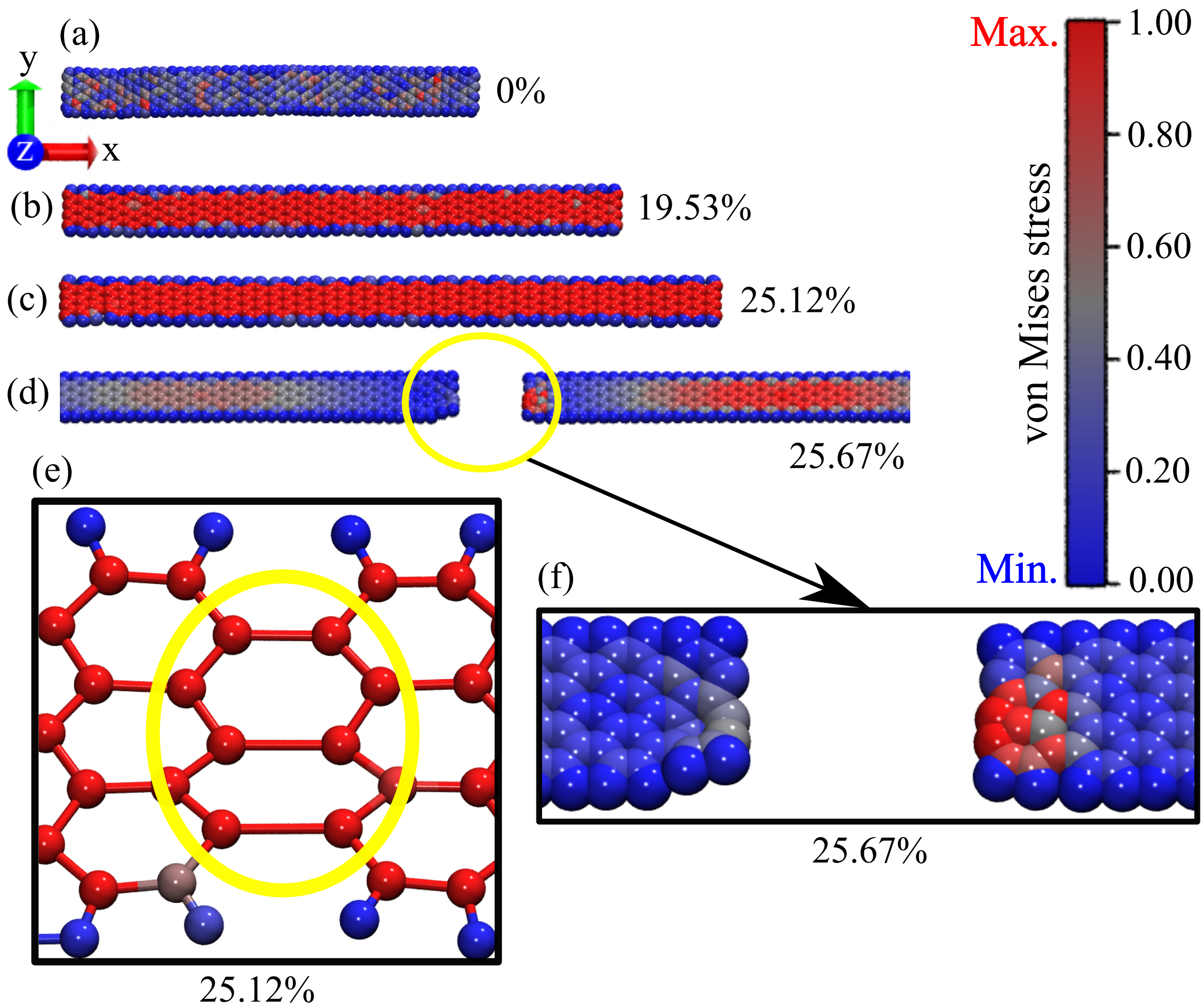

FIG. S3. **Representative MD snapshots illustrating the tensile stretching process of an AC-RNR with a width of W_1 = 9.12 Å. Please refer to Table S1 for additional information.** (a) initial structure of the RNR, at 0% strain. (b) and (c) structures of the RNR at strain values of 19.53% and 25.12%, respectively. (d) Structure and strain value at which the fracture happens. (e) A zoomed-in view of the specific C–C bonds just before rupture (at 25.12% of strain load). (d) Another zoomed-in view showing the breakage of the C–C bonds at 25.67% of strain (fully fractured). In the sidebar on the right side of the images, regions depicted in red indicate areas of high-stress accumulation, whereas those in blue indicate low-stress levels.

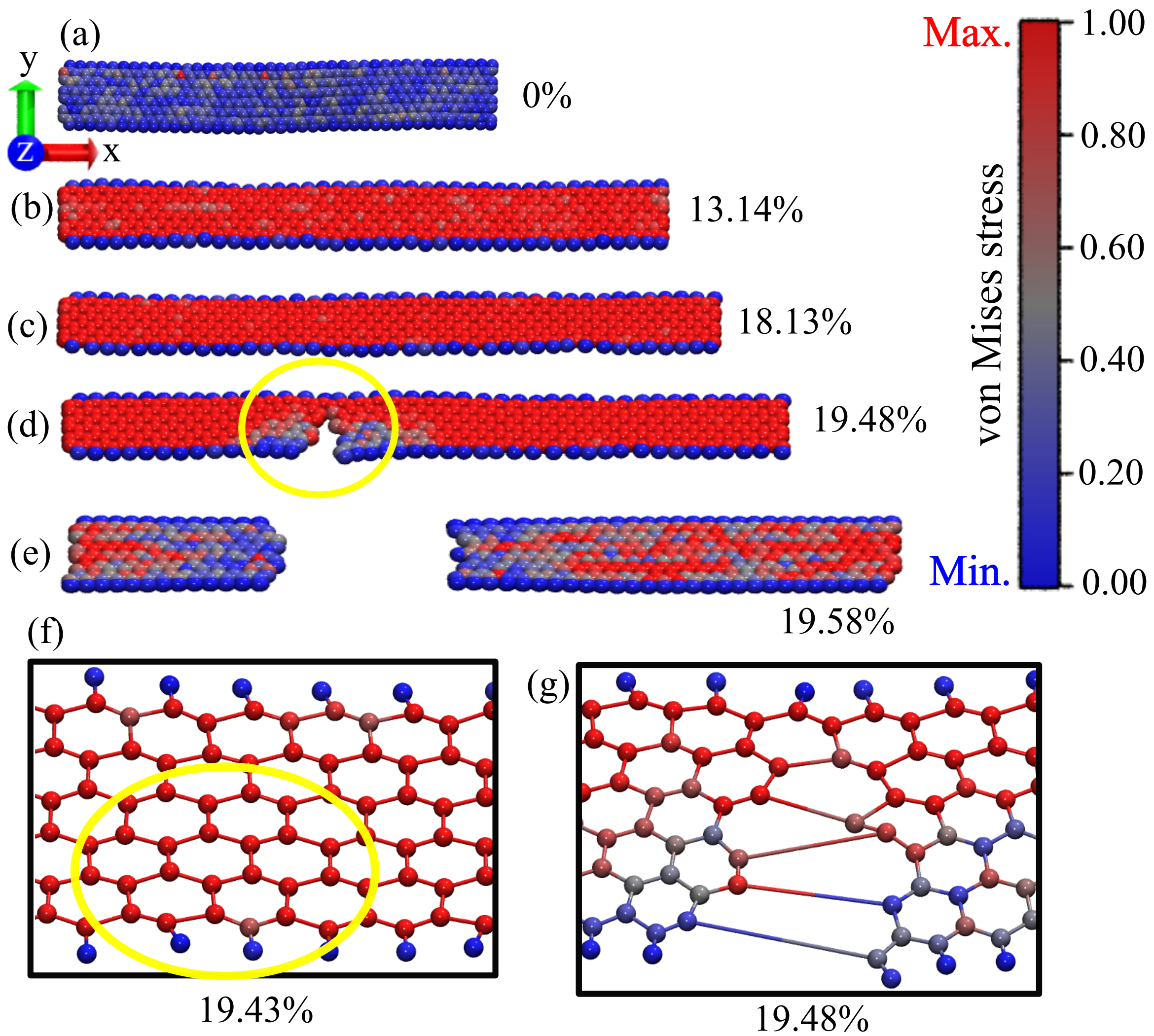

FIG. S4. **Representative MD snapshots illustrating the tensile stretching process of an ZZ-RNR with a width of W_1 = 13.14 Å. Please refer to Table S1 for additional information.** (a) initial state of the RNR, at 0% strain. (b) and (c) structures of the RNR at strain values of 13.14% and 18.13%, respectively. (d) Structure and strain value at which the fracture starts. (e) Structure and strain value at which the fracture happens. (f) A zoomed-in view of stretched C–C bonds. (g) A zoomed-in view of the breakage of some C–C bonds at 19.48% of strain load (beginning of the mechanical failure). In the sidebar on the right side of the images, regions depicted in red indicate areas of high-stress accumulation, whereas those in blue indicate low-stress levels.

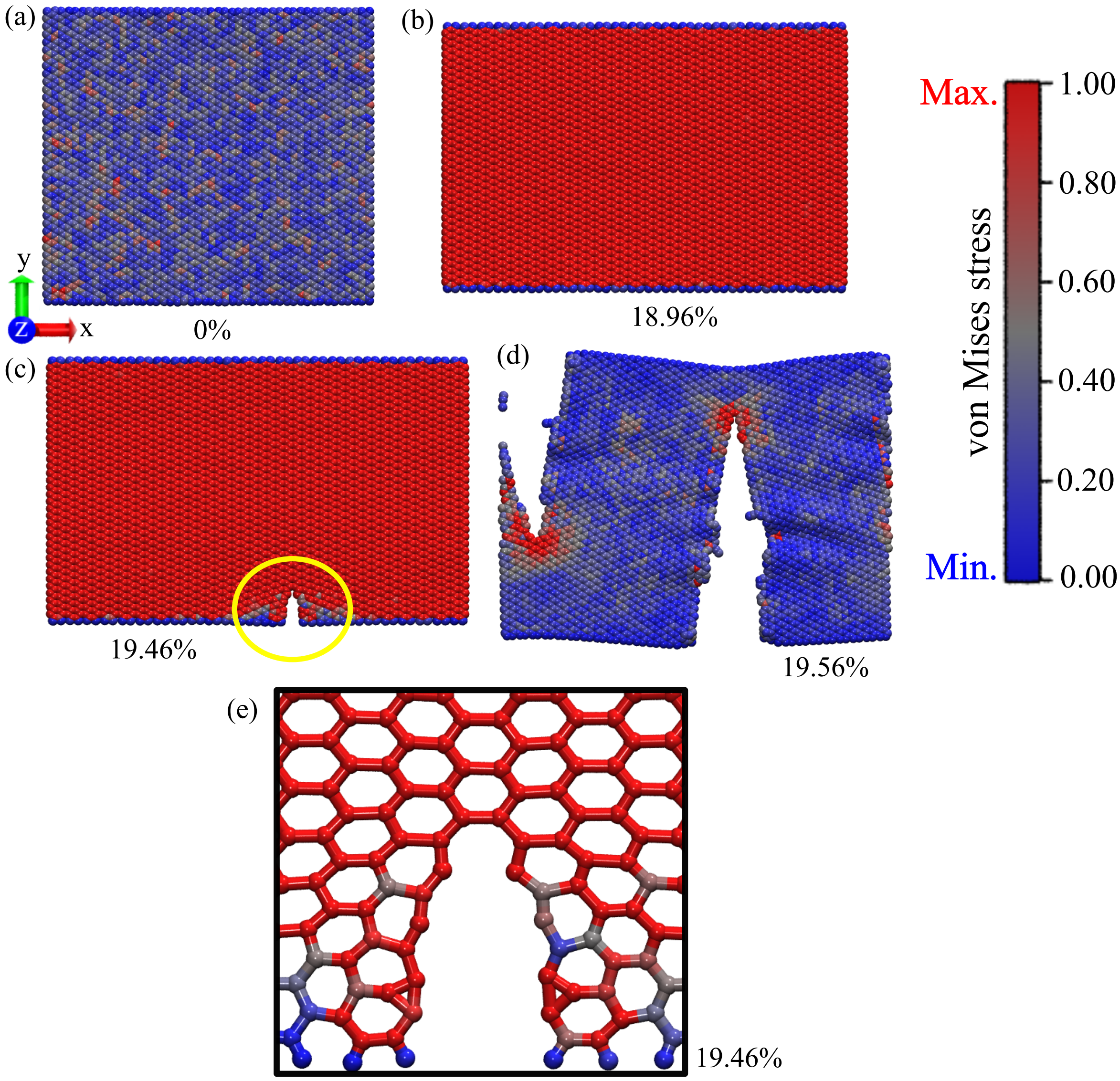

FIG. S5. **Representative MD snapshots illustrating the tensile stretching process of an AC-RNR with a width of W_1 = 90.42 Å. Please refer to Table S1 for additional information.** (a) initial state of the RNR, at 0% strain. (b) and (c) structures of the RNR at strain values of 18.96% and 19.46%, respectively. (d) Structure and strain value at which the fracture is advanced. (e) A zoomed-in view of the crack at 19.46% of strain. In the sidebar on the right side of the images, regions depicted in red indicate areas of high-stress accumulation, whereas those in blue indicate low-stress levels.

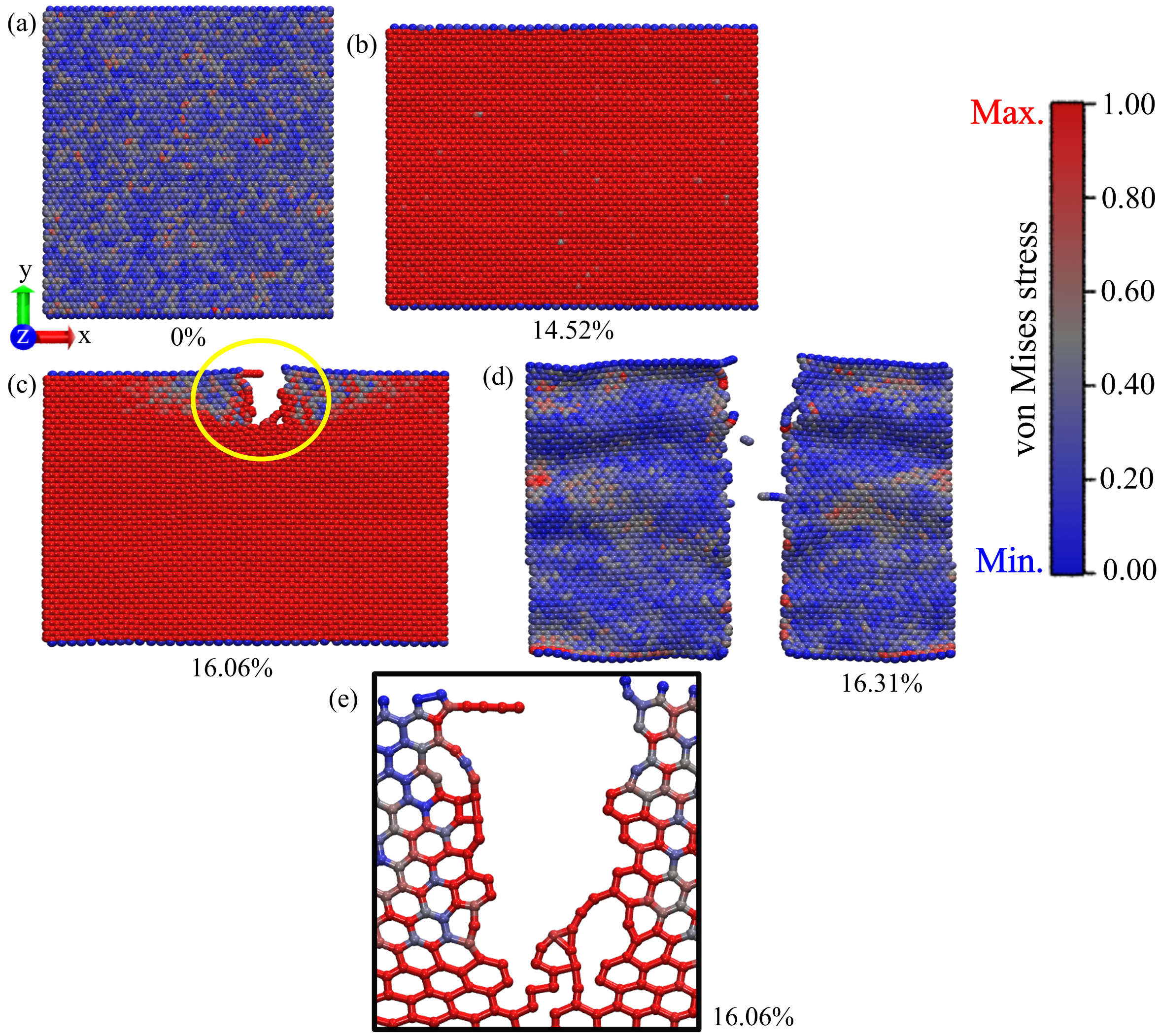

FIG. S6. **Representative MD snapshots illustrating the tensile stretching process of an ZZ-RNR with a width of $W_1 = 106.08$ Å. Please refer to Table S1 for additional information.** (a) initial state of the RNR, at 0% strain. (b) and (c) structures of the RNR at strain values of 14.52% and 16.06%, respectively. (d) Structure and strain value at which the fracture happens. (e) A zoomed-in view of the crack at 16.06% of strain. In the sidebar on the right side of the images, regions depicted in red indicate areas of high-stress accumulation, whereas those in blue indicate low-stress levels.

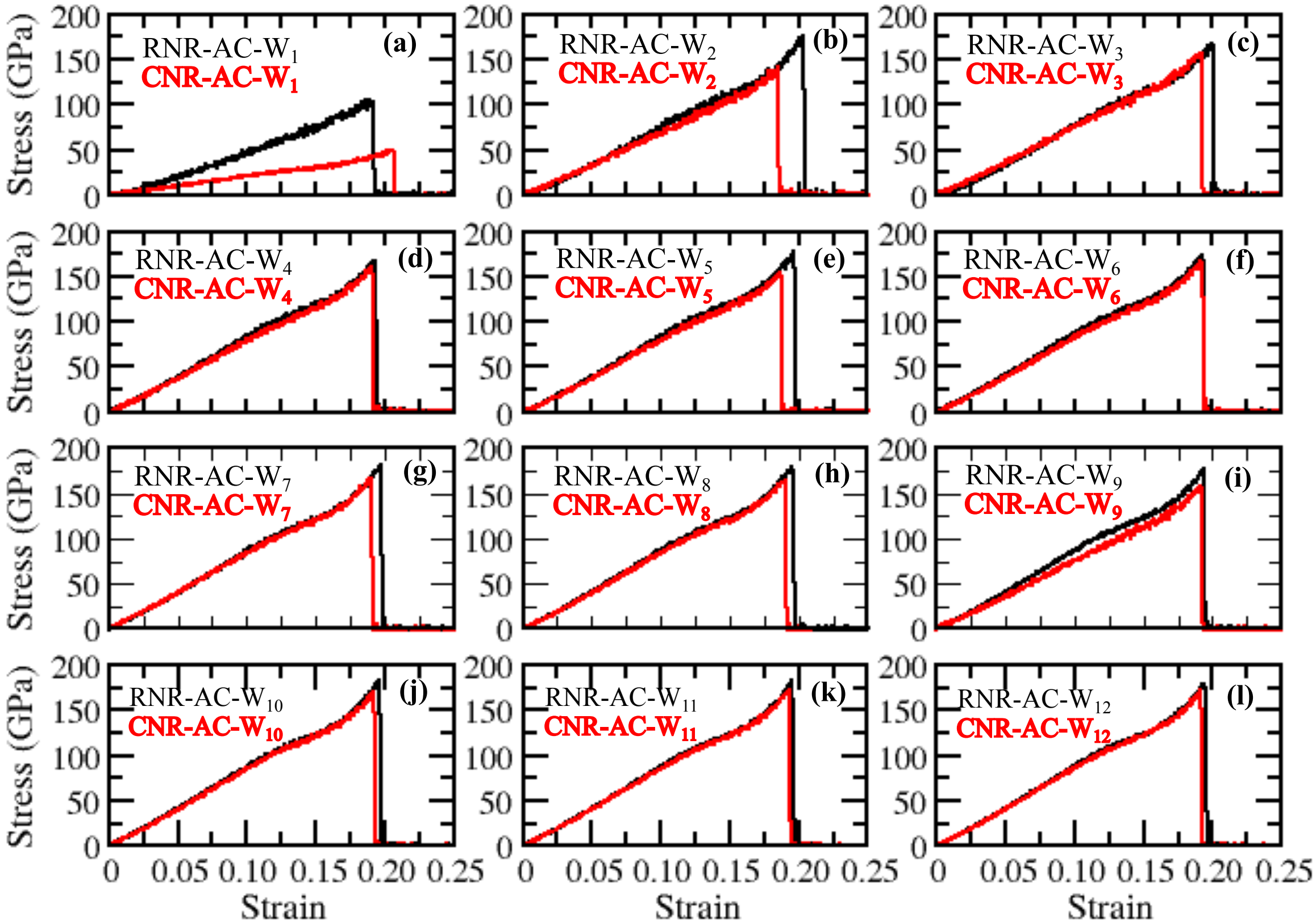

FIG. S7. **Sets of stress-strain curves of AC-RNRs and AC-CNRs for each width value. The values of width are given in Table S1.** (a) W_1, (b) W_2, (c) W_3, (d) W_4, (e) W_5, (f) W_6, (g) W_7, (h) W_8, (i) W_9, (j) W_{10}, (k) W_{11} and (l) W_{12}.

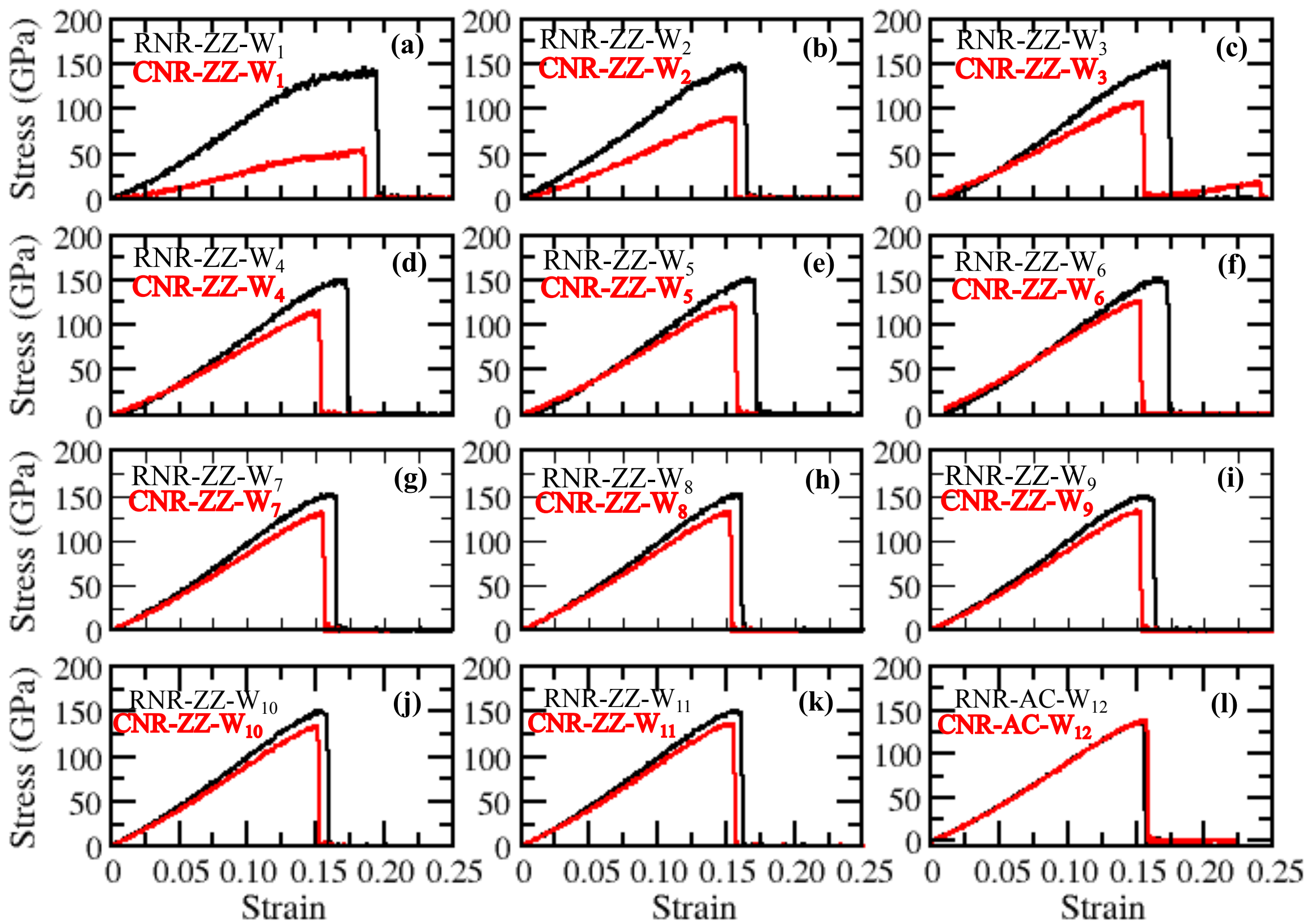

FIG. S8. **Sets of stress-strain curves of ZZ-RNRs and ZZ-CNRs for each width value. The values of width are given in Table S1.** (a) W_1, (b) W_2, (c) W_3, (d) W_4, (e) W_5, (f) W_6, (g) W_7, (h) W_8, (i) W_9, (j) W_{10}, (k) W_{11} and (l) W_{12}.

TABLE S1. Information about simulated AC-RNRs and ZZ-RNRs: width (Å) and number of atoms.

System	Width (Å)	Number of Atoms
AC-RNR-W_1	9.12	432
AC-RNR-W_2	16.46	720
AC-RNR-W_3	23.84	1008
AC-RNR-W_4	31.21	1296
AC-RNR-W_5	38.84	1584
AC-RNR-W_6	46.21	1872
AC-RNR-W_7	53.58	2160
AC-RNR-W_8	60.95	2448
AC-RNR-W_9	68.31	2736
AC-RNR-W_{10}	75.68	3024
AC-RNR-W_{11}	83.06	3312
AC-RNR-W_{12}	90.42	3600
System	Width (Å)	Number of Atoms
ZZ-RNR-W_1	13.34	574
ZZ-RNR-W_2	21.85	902
ZZ-RNR-W_3	30.36	1230
ZZ-RNR-W_4	38.87	1558
ZZ-RNR-W_5	47.38	1886
ZZ-RNR-W_6	55.88	2214
ZZ-RNR-W_7	64.39	2542
ZZ-RNR-W_8	72.90	2870
ZZ-RNR-W_9	81.52	3198
ZZ-RNR-W_{10}	90.04	3526
ZZ-RNR-W_{11}	97.56	3854
ZZ-RNR-W_{12}	106.08	4182

TABLE S2. Elastic properties of AC and ZZ RNRs at room temperature. Young's Modulus (Y_M) are given in gigapascals (GPa), Ultimate Tensile Strength (UTS) in GPa and critical strain (ϵ_C) in percentage (%). The estimated uncertainties in the UTS and ϵ_C are 5 GPa and 0.05 %, respectively. For an elucidation of the uncertainties, refer to the Methods section of the main text.

System	Y_M (GPa)	UTS (GPa)	ϵ_C (%)
AC-RNR-W_1	679.91 ± 19.70	108.84	19.34
AC-RNR-W_2	772.23 ± 19.55	175.38	20.44
AC-RNR-W_3	795.78 ± 17.24	168.22	20.13
AC-RNR-W_4	843.19 ± 13.65	167.42	19.46
AC-RNR-W_5	856.19 ± 3.42	177.43	19.83
AC-RNR-W_6	869.24 ± 2.35	174.41	19.46
AC-RNR-W_7	871.67 ± 2.23	181.36	19.89
AC-RNR-W_8	880.32 ± 8.63	179.22	19.69
AC-RNR-W_9	884.17 ± 7.30	175.63	19.49
AC-RNR-W_{10}	885.81 ± 8.22	182.44	19.76
AC-RNR-W_{11}	893.98 ± 6.10	181.94	19.69
AC-RNR-W_{12}	894.29 ± 5.57	178.57	19.58
ZZ-RNR-W_1	910.84 ± 14.72	141.46	19.58
ZZ-RNR-W_2	963.17 ± 15.00	148.91	17.50
ZZ-RNR-W_3	973.76 ± 10.99	152.24	17.50
ZZ-RNR-W_4	974.13 ± 10.82	150.17	17.38
ZZ-RNR-W_5	984.00 ± 16.17	151.23	17.26
ZZ-RNR-W_6	1023.60 ± 11.69	151.84	17.43
ZZ-RNR-W_7	1043.70 ± 12.92	152.40	16.55
ZZ-RNR-W_8	1056.70 ± 9.72	151.24	16.21
ZZ-RNR-W_9	1068.20 ± 8.20	150.85	16.38
ZZ-RNR-W_{10}	1075.30 ± 6.52	151.95	15.90
ZZ-RNR-W_{11}	1080.45 ± 6.48	151.34	16.27
ZZ-RNR-W_{12}	1082.15 ± 6.31	138.84	15.94
